# Habitat selection in a fluctuating ground squirrel population: Density‐dependence and fitness consequences

**DOI:** 10.1002/ece3.9241

**Published:** 2022-08-29

**Authors:** Jaclyn R. Aliperti, Kimberly Jenderseck, Dirk H. Van Vuren

**Affiliations:** ^1^ Department of Wildlife, Fish, and Conservation Biology University of California Davis CA USA; ^2^ Rocky Mountain Biological Laboratory Crested Butte CO USA

**Keywords:** home range, population dynamics, settlement decision, space use, survival

## Abstract

Investigating individual‐based habitat settlement decisions is a central theme in ecology, yet studies that quantify density‐dependent habitat selection or tie fitness to resource selection decisions remain rare. We quantified habitat selection in golden‐mantled ground squirrels (*Callospermophilus lateralis*) across two spatial scales (home‐range placement, and occurrence within the home range) by using 11 consecutive years of data on individual space use, and we used resource selection functions and multilevel modeling to address how habitat preferences may be influenced by density or linked to fitness outcomes. Squirrels preferred dry meadow over other habitat types (wet meadow, aspen, spruce, and willow) at both spatial scales. Squirrels were more likely to use dry meadow that contained shorter vegetation and vision‐enhancing prominences such as rocks (“perches”). The use of dry meadow at each scale was not influenced by changes in density. The use of dry meadow did not lead to increased litter size, pre‐hibernation mass, or survival. However, squirrels that experienced a greater number of perches or lower local densities had higher survival rates. Our results suggest that a lack of visual obstruction, probably facilitating detection of predators, drives habitat selection in this system. Surprisingly, squirrels maintained their preference for dry meadow as density increased, and they experienced reduced survival as a result. This work furthers our understanding about the causes and consequences of changes in habitat use, informing wildlife management and conservation.

## Graphical abstract

We quantified habitat selection in golden‐mantled ground squirrels (Callospermophilus lateralis) across two spatial scales (home range placement, and occurrence within the home range) by using 11 consecutive years of data on individual space use, and we used resource selection functions and multilevel modeling to address how habitat preferences may be influenced by density or linked to fitness outcomes. Surprisingly, squirrels maintained their preference for dry meadow as density increased, and they experienced reduced survival as a result. This work furthers our understanding about the causes and consequences of changes in habitat use, providing insight to wildlife management and conservation.

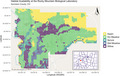

## 1 INTRODUCTION

Investigating individual‐based habitat settlement decisions is a central theme in ecology (Morris, 2003), having ties to fundamental concepts such as niche theory (Hutchinson, 1957; Vandermeer, 1972) and optimal foraging (Charnov, 1976; Fretwell & Lucas, 1969; MacArthur & Pianka, 1966). In nature, resources are typically finite and nonuniformly distributed (Morris, 1989; Paterson & Blouin‐Demers, 2018), and habitat selection often depends on predation risk (“landscape of fear”; Brown & Kotler, 2004, Laundré et al., 2010), including physical structure that affects risk (Kohl et al., 2018; Smith et al., 2019). Habitat selection, therefore, dictates the distribution of individuals across space and time (van Beest et al., 2014), which influences access to key resources such as preferred food items or cover from predators (Chalfoun & Martin, 2007; Clark, 1994). Hence, individuals often differ in performance, or fitness (e.g., survival, reproduction), across habitat types (Gaillard et al., 2010; Mcloughlin et al., 2007; Paterson & Blouin‐Demers, 2018), and habitat preferences are shaped by fitness benefits (Chalfoun & Martin, 2007; Uboni et al., 2017).

It is well known that habitat‐performance relationships are influenced by density, as an individual's realized fitness is decreased by intraspecific competition (Morris, 2003; Uboni et al., 2017). The ideal‐free distribution (IFD) theorizes that animals take this into account when self‐assorting among habitat types; as population density increases in high‐quality habitat and competition for limited resources becomes intense, animals preferentially settle in lower quality habitat, where they are equally likely to survive or reproduce due to lack of competition (Fretwell & Lucas, 1969). Some studies criticized the validity of the underlying assumptions, noting that factors such as differential access to resources among individuals (Conradt et al., 1999; Parker & Sutherland, 1986), predation risk (Garshelis, 2000; Morris, 1989; Thompson & Gese, 2012), lack of accurate information (Abrahams, 1986; Hemingway et al., 2018), movement costs (Abrahams & Labelle, 2020; Matsumura et al., 2010), or ecological traps (Robertson et al., 2013; Schlaepfer et al., 2002) might dominate settlement decisions. Nonetheless, most criticisms remain untested in the field, in part because accurate assessments of habitat quality should involve calculations of animal performance (Mosser et al., 2009; Van Horne, 1983), yet few investigations tie direct measurements of survival or reproduction to resource selection decisions by individual animals (Chalfoun & Martin, 2007; Gaillard et al., 2010; Pulliam & Danielson, 1991). Modeling approaches such as the resource selection function, which calculates habitat use in proportion to availability to deduce habitat preference (Boyce & Mcdonald, 1999; Manly et al., 2002), are increasingly paired with measures of fitness to inform wildlife management (Gaillard et al., 2010; McLoughlin et al., 2006). However, studies that quantify the effect of density on habitat selection in free‐ranging animal populations remain rare (McLoughlin et al., 2010; Morris & MacEachern, 2010; van Beest et al., 2014).

In addition to varying as a function of density, habitat selection patterns (and associated fitness consequences) may differ across spatial scales (Dupke et al., 2017; Gaillard et al., 2010; Johnson, 1980). For example, habitat choices by moose (*Alces alces*) at the landscape scale were driven by food availability, while finer‐scale resource use was associated with refugia from human‐induced risk (Herfindal et al., 2009); conversely, woodland caribou (*Rangifer tarandus*) selected habitat based on avoiding predation on broader spatial scales, while selecting habitat associated with better forage at finer scales (Rettie & Messier, 2000). Hence, animals continuously experience a variety of ecological tradeoffs, causing individuals to actively select habitat that best meets their current needs at each of a range of spatial scales (Dussault et al., 2005). Johnson (1980) categorized these scales into hierarchical “orders”: the broad‐scale geographic distribution of a species, placement of an individual's home range (Burt, 1943; Powell & Mitchell, 2012), use of certain areas within the home range, and selection for specific food items within those areas. The use of habitat types or resources becomes selective when it is disproportionate to its availability at each spatial scale; thus, it is possible that a species may be selecting for a given resource at only a subset of the spatial scales in which it is used (Johnson, 1980; Mayor et al., 2009).

Given that habitat types that differ in availability across spatial scales may offer important resources that meet separate fitness needs (Beyer et al., 2010; Gaillard et al., 2010) and that density dependence may affect the use of selected habitat types (Mayor et al., 2009; Morris, 1989), habitat preference is complex and context‐dependent. As such, it is critical to define appropriate scales of study (reviewed by Mayor et al., 2009, McGarigal et al., 2016), ideally at several spatial scales and for multiple fitness parameters (Chalfoun & Martin, 2007; Gaillard et al., 2010). Moreover, wildlife management is improved through quantification of fitness–habitat relationships over large spatial (i.e., home range as opposed to feeding site) and temporal (i.e., across multiple years as opposed to a single season) scales and during periods of high and low density, which allows for conservation of features that are critical to individual survival and reproduction (Boyce, 2006; McLoughlin et al., 2006; van Beest et al., 2014). However, long‐term studies that use direct, individual‐based measures of fitness to better understand the consequences of selecting habitat at scales larger than the feeding site are uncommon because of practical difficulties in data collection (Mcloughlin et al., 2007).

We used 11 consecutive years of detailed information on individual space use to investigate the causes and consequences of habitat selection in the golden‐mantled ground squirrel (GMGS; *Callospermophilus lateralis*) at a high elevation site in Colorado. Our study captured a period of population flux, resulting in a range of population densities. We quantified habitat selection across two ecologically relevant spatial scales (home‐range placement, and use within the home range), assessed the effect of density on habitat selection, and evaluated several fitness outcomes of the habitat selection decision. We expected that habitat selection would be influenced by density, and that the habitat selection decision would have fitness consequences.

## 2 MATERIAL AND METHODS

### 2.1 Study system

GMGS occur in mid‐ to high‐elevation mountainous regions in the western United States and Canada (Bartels & Thompson, 1993; McKeever, 1964). As a small‐bodied species (150–300 g), GMGS are presumed to be asocial (Armitage, 1981; Michener, 1983), and limited research suggests that GMGS are generally agonistic toward conspecifics (Ferron, 1985). GMGS appear to use a variety of habitat types, such as conifer forest, chaparral, sagebrush, and mountain meadows, but habitat preference is poorly known (Bartels & Thompson, 1993; McKeever, 1964; Shick et al., 2006).GMGS hibernate in their burrows for much of the year, with populations at higher elevations spending up to twice as long hibernating than those at lower elevations (Bronson, 1979; McKeever, 1964). Females mate shortly after emerging from hibernation (Wells et al., 2017), undergo gestation for approximately 28 days, and give birth to one litter of 1–8 pups per year (4.8, on average; Kneip et al., 2011, Wells & Van Vuren, 2017). The mean sex ratio of offspring is 1:1, and pups are weaned after about 30 days of lactation, whereupon they emerge from their natal burrow (Wells & Van Vuren, 2017).

We studied GMGS in Gothic, Colorado, at the site of the Rocky Mountain Biological Laboratory (RMBL), located in the East River Valley, Gunnison County (38°57′ N, 106°59′ W), from 1996 through 2006. Given the high elevation at RMBL (2900 m), squirrels enter hibernation during late August, and they do not emerge until the approximate time of snowmelt, usually during May (Hostetler et al., 2012; Kneip et al., 2011). GMGS are primarily herbivorous, with a diet comprised mostly of forbs and fungi (Haufler & Nagy, 1984; McKeever, 1964), though arthropods are sometimes consumed, and opportunistic predation of small mammals and nestling birds has been recorded (McKeever, 1964; Troy & Conover, 2019). The main predators of GMGS at RMBL are red foxes (*Vulpes vulpes*), long‐tailed weasels (*Mustela frenata*), and short‐tailed weasels (*Mustela erminea*) (Kneip et al., 2011).

The 16‐ha study area was bounded by the East River on the west, Copper Creek on the south, and aspen (*Populus tremuloides*) forest on the north and east. The study area was a mosaic of macrohabitats, mostly consisting of dry meadow, interspersed with patches of wet meadow, groves of aspen and spruce (*Picea engelmannii*), and stands of willow (*Salix* spp.). Dry meadow consisted of grasses (e.g., *Bromus inermis*, *B. polyanthus*) and low‐growing forbs, such as dandelion (*Taraxacum officinale*), aspen sunflower (*Helianthella quinquenervis*), and cinquefoil (*Potentilla* spp.). Dry meadow was sparse in places and structurally open, and contained patchily distributed rocks. Wet meadow consisted of dense stands of tall forbs that often reached 1 m in height, including Colorado false hellebore (*Veratrum tenuipetalum*), cow parsnip (*Heracleum maximum*), and subalpine larkspur (*Delphinium barbeyi*). Aspen groves had an open understory of scattered patches of tall forbs, such as cow parsnip and perennial herbs (e.g., *Ligusticum porteri* and *Osmorhiza occidentalis*). Spruce groves had dense canopies, creating relatively shaded understories largely devoid of herbaceous vegetation, but including some shrubs, such as whortleberry (*Vaccinium myrtillus*) and currant (*Ribes montigenum*). While aspen and spruce habitats contained structurally different understories, both habitat types included downed wood in the form of branches and stumps. Willow stands were dense and consisted of shortfruit willow (*Salix brachycarpa*), Drummond's willow (*S. drummondiana*), and Booth's willow (*S. boothii*). Slope aspect ranged from south to west, and slopes were gentle (<5°), except for a few localities where slopes reached 15°. Wet meadow and willow habitats occurred mostly on gentle slopes; otherwise, habitat type was not associated with slope or slope aspect.

Fieldwork was conducted from June through August each year. During early June, we conducted an annual census by trapping all GMGS in the study area using Tomahawk live‐traps (Model 201). Additionally, we trapped pups when they first emerged from their natal burrows, usually late June to mid‐July. Captured squirrels received numbered ear tags and unique dye marks (Nyanzol D, Greenville Colorants) on their fur for visual identification, and sex, body mass, and reproductive status were recorded (Wells & Van Vuren, 2017). Squirrels were re‐trapped periodically to renew dye marks and determine mass and reproductive status. Trapping continued until visual observations indicated no unmarked squirrels. Squirrel locations in the study area were determined visually using binoculars, with animal identity based on unique dye marks. Individuals were diurnal and readily observable when not underground. We used instantaneous scan sampling, in which we recorded the identity and location of each squirrel in view at 1‐min intervals. Squirrel locations were determined based on a grid map of the study area with 7‐m × 7‐m cells. Habitat features allowed accurate association of a squirrel's location with a specific grid cell. Squirrels in our study area frequently encountered humans and rarely changed their behavior due to our presence from the observation distances we used. If a squirrel did react, as indicated by either fleeing from the observer or remaining motionless and staring at the observer, those observations were discarded. A given sampling bout continued until squirrels left the area or entered a burrow, usually <10 min. We observed all sections of the study area at least twice per day, once during the morning and once during the afternoon. While we observed squirrels from a given location, other sections of the study area were out of view; hence, we rotated among different sections of the study area at varying times of day to promote an even distribution of sampling effort. Nonetheless, some individuals were more active or easily seen than others; thus, we directed more effort toward observing underrepresented squirrels in the population when appropriate. We combined observations for each resident adult (≥1‐year old) female each year.

Macrohabitat type was determined by visiting each grid square during the summers of 2000 and 2001 and visually identifying the macrohabitat type that comprised the greatest percent cover of that square. Some grid squares included buildings or gravel roads, totaling 7.7% of all grid squares. Squirrels were excluded from buildings, and squirrels never used roads except when in transit; hence, if buildings or roads comprised the majority of a given grid square, then that grid square was reassigned to the next most common macrohabitat type present.

A lack of visual obstruction appears to be important to ground squirrels in detecting predators (Blumstein et al., 2006; McGrann et al., 2014), and it might be especially important for a small‐statured species such as GMGS. Microhabitat features that potentially influenced visual obstruction varied among the five macrohabitats that we studied, but only in dry meadow were we able to measure such microhabitat features reliably and consistently; there was often no herbaceous layer in spruce forest, and visibility from a squirrel perspective was near zero at all heights above the ground in wet meadow and willow habitat. Hence, for each grid square that supported dry meadow, we determined vegetation height and cover. Vegetation height was measured at five locations per grid square (in the center and toward each of four corners), then averaged and assigned to one of four categories: 0–25, 26–50, 51–75, and >75 cm. Vegetation cover for the grid square was assessed by ocular estimate in four categories: 0%–25%, 26%–50%, 51%–75%, and 76%–100%. In addition, GMGS often sat on prominences such as rocks or stumps, presumably because of the enhanced field of vision (Machutchon & Harestad, 1990). Hence, we recorded the number of suitable “perches”; a perch was considered to be suitable when it was taller than the maximum height of vegetation within a given grid square. We counted the number of suitable perches in each grid square up to 10, and assigned the number 11 to those grid squares with more than 10 perches. Microhabitat features were measured from late June until early August, after maximum vegetation growth was reached and before vegetation began to senesce.

We assessed density effects and fitness consequences during 1996–2006, representing 5 years before and after habitat characterizations were completed; vegetation at both the macrohabitat and microhabitat level was consistent during that time period. To estimate population density, we recorded the total number of adult females present in the study area at the beginning of the active season each year. However, because the occurrence of female GMGS in our study area tended to be in clusters, which might influence the density experienced by each female (Wells & Van Vuren, 2017), we also investigated the effects of local density on habitat selection. We calculated the number of neighboring adult female home ranges that overlapped with a focal female's home range in a given year, and we used this number as a proxy for local density (Efford, 1996; Sanchez & Hudgens, 2015; Woodworth et al., 2017).

Fitness measures for GMGS were calculated from demographic data collected during annual trapping. Survival was determined annually, based on presence during the early‐June census from 1 year to the next; dispersal of adult females out of the study site during this period of time was rare. Reproductive success can be measured by reproductive status (whether or not a female bred) or by litter size (number of offspring produced per female that bred). Most females (67%–100%) reproduced each year during our study, precluding analysis of reproductive status, so we instead focused on litter size of reproductive females. Litter size in GMGS might be influenced by stored fat from the previous summer (capital breeding) or resource acquisition during reproduction (income breeding; Stearns, 1992). Consequently, we investigated relationships between habitat use and litter size during both the same year and the year after habitat use was measured. Litter size might have been influenced by infanticide or predation; however, we observed no evidence of infanticide, and litters that suffered predation before litter size was known were excluded from analysis. Maternity was assigned when a litter emerged at the female's burrow, reinforced by observations within 48 h of pup emergence, when littermate pups typically engage in affiliative interactions, such as nose‐greets, with only their mother and each other (Ferron, 1985). Maternity and litter assignments were confirmed by genetic analysis (Wells & Van Vuren, 2017). Females that were reproductive during a given year, as indicated by swollen nipples during June, but whose litters were likely predated and hence never observed, were assigned the mean number of offspring produced by females who bred that year (*n* = 4 for same‐year analysis, *n* = 6 for next‐year analysis). For a hibernating species such as GMGS, ample fat reserves at entry into hibernation should promote overwinter survival (Dark, 2005). Hence, as an additional fitness measure, we estimated pre‐hibernation body mass for each female based on mass at her last capture that summer. We regressed mass at last capture of females against time, and used the slope of the regression (i.e., the population‐level rate of increase in late season body mass) to estimate the mass of each female on 31 August; females entered hibernation in late August, and growth of adult GMGS is generally linear during the second half of the active season (Phillips, 1984).

### 2.2 Multi‐scale habitat selection

Grid square locations were registered in a spatial database of the study area in ArcGIS v10 (ESRI, 2018). GMGS sometimes moved across multiple grid squares comprising more than one habitat during a sequence of scan samples. However, they often remained in the same general location from 1 min to the next, resulting in the possibility of spatial autocorrelation in observations of squirrel locations. We reduced spatiotemporal autocorrelation in the dataset by including only the first observation when an individual was recorded in the same grid square more than once within a 10‐min period. We delineated an annual home range for each squirrel by calculating 95% minimum convex polygons through package adehabitatHR (Calenge, 2006) in the R software (version 3.6.1; R Core Team, 2019). We included only females for which we recorded at least 55 independent (autocorrelation‐corrected) observations within a given year, which surpassed sample sizes used to calculate squirrel home ranges in other studies (Jesmer et al., 2011; Romeo et al., 2010; Wauters et al., 2007). In addition, we included only females that were observed across at least 20 different calendar days each year.

Macrohabitat selection was measured on two spatial scales: habitat composition of the home range compared to that available across the study area (second‐order selection), and habitat types used compared with those available within an individual's home range (third‐order selection; Johnson, 1980). Habitat composition of each home range (second‐order) was based on habitats assigned to those grid squares within the home range, and use within each home range (third‐order) was based on the proportional distribution of observations of the squirrel among habitats. All squirrels shared the same available habitat for second‐order selection, but habitat availability was unique to each individual for third‐order selection. We assessed habitat selection by calculating selection ratios (Manly et al., 2002) for each animal in each habitat type, and we used chi‐square goodness of fit tests to compare the proportion of used versus available habitat for each habitat type across all individuals.

To assess the effect of microhabitat features on habitat selection in dry meadow habitat, we ran a generalized linear mixed effects model (GLMM) with a Poisson error distribution and log link that examined counts, or total observations, in each available grid square of dry meadow as a function of microhabitat characteristics of those grid squares. We held individuals, grid locations, and years of study as random effects to account for repeated measures and the possibility of inherent differences among grids or interannual variation in environmental conditions. Environmental variables such as temperature or sun intensity might influence squirrel movements, but such data were either unavailable or could not be paired with our data on squirrel locations. The presence of predators might also influence movements; predation intensity varied annually, based on whether foxes or weasels were active that summer, and that effect is captured by the inclusion of year in all models. We used R packages lme4 (Bates et al., 2015) and glmmTMB (Brooks et al., 2017) to fit all mixed effects models. We used glmmTMB when convergence was an issue for lme4 and we wanted to maximize the number of biologically relevant variables that could explain inherent variation in the data. The glmmTMB package possesses higher speed and flexibility than the lme4 package when running generalized mixed effects models (Brooks et al., 2017), and its interface is designed to be similar to the lme4 package (Bates et al., 2015). For cases in which a given model converged in lme4, we achieved nearly identical results when we ran that same model in glmmTMB. Hence, the use of both packages did not influence our overall results.

### 2.3 Density effects and fitness consequences

We found that GMGS selected only for dry meadow habitat; hence, analyses investigating density‐dependence and fitness consequences focused only on use of dry meadow. We assessed the effects of density on use of dry meadow by fitting separate models for each level of density (population and local) at each spatial scale (second‐order and third‐order). The second‐order models analyzed the proportion of a squirrel's home range comprised of dry meadow habitat in relation to density and age, and the third‐order models analyzed the proportion of observations in a squirrel's home range that was within dry meadow in relation to density and age. We included age, as well as an interaction between age and density, as fixed effects because the habitat selection decision might be most evident at the time of settlement; in our GMGS population, dispersal begins late in the summer of birth and females typically establish residency by 1 year of age. Hence, we coded age as a binary variable that categorized each squirrel as being either a yearling or older. All models were logistic GLMMs with a binomial error distribution and logit link that weighted the outcome by the total number of grid squares in a squirrel's home range (second‐order model) or the total number of observations recorded for each squirrel (third‐order model) and that included individuals and years as random effects.

We fit a series of GLMMs to examine the effects of multi‐scale use of dry meadow (percent of home range, percent of observations within home range) on annual survival, litter size, and pre‐hibernation body mass. The relationship between habitat use and each fitness outcome can be confounded by other variables thought to influence survivorship or reproductive success. Hence, we also included density and age as predictors in all of our fitness models, pre‐hibernation mass as a predictor in our survival and next‐year reproductive fitness models, and emergence mass as a predictor in our same‐year reproductive fitness model; emergence mass was recorded as body mass during the first trapping event of the season, with the body mass of each female on 1 June estimated via linear regression as previously described for estimating pre‐hibernation mass. We censored three deaths from collisions with vehicles from the dataset and used a logistic regression with a binomial error distribution and logit link to model the relationship between habitat use and annual survival. To assess the effects of habitat use on this‐ and next‐year litter size, we modeled data with a Poisson error distribution and log link. To investigate the relationship between habitat use and pre‐hibernation body mass, we fit a log linear model with a Gaussian distribution. We scaled and centered all continuous predictor variables and accounted for individuals and years of study as random effects in all fitness models. We iteratively removed non‐significant predictors and compared Akaike information criterion corrected for small sample size (AICc) to choose the most parsimonious models that minimized prediction error (Table S1).

## 3 RESULTS

**FIGURE 1:**
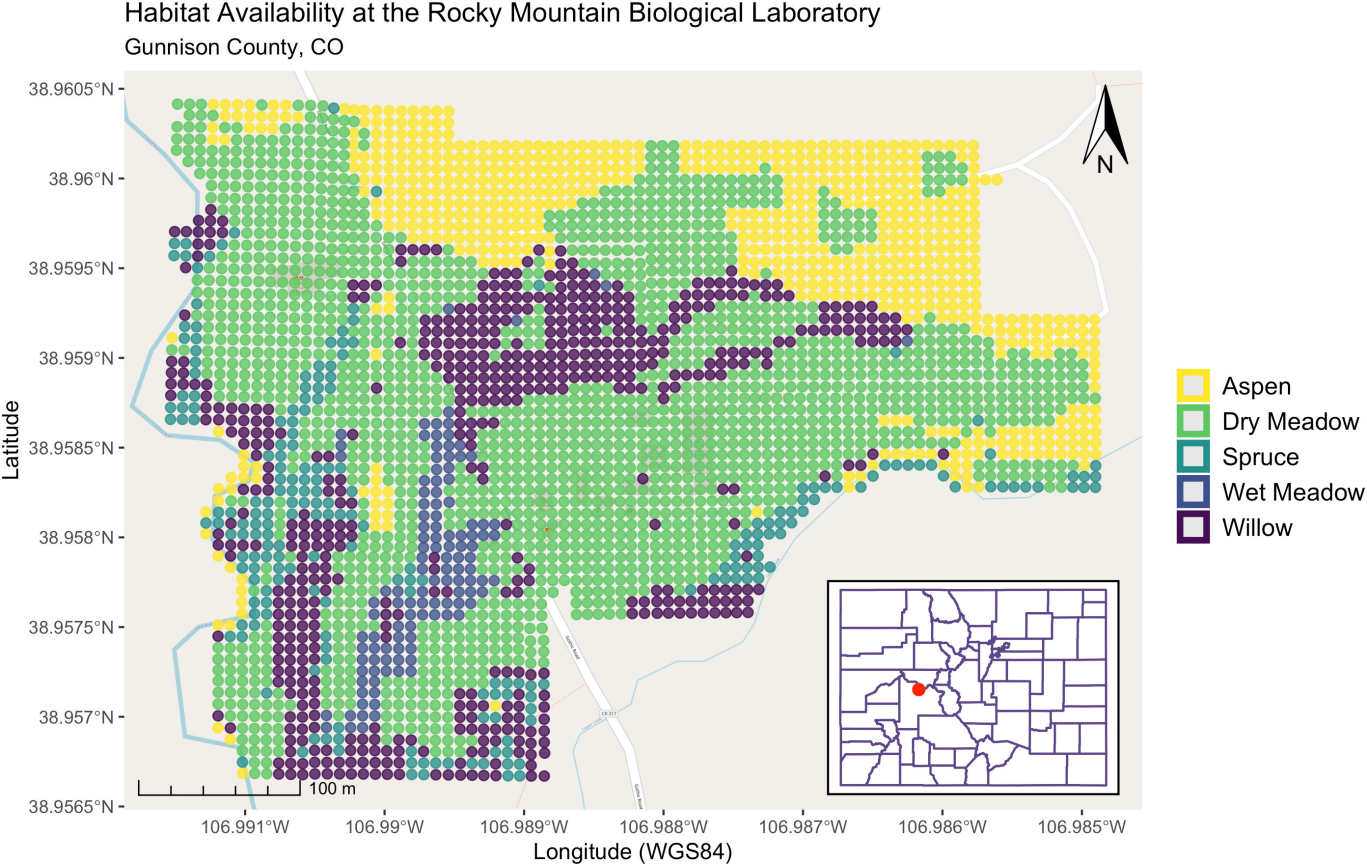
Habitat map of the study area, located at the Rocky Mountain biological laboratory, Gunnison County, Colorado. The site is delineated by a 7 × 7 m grid system, with each grid cell assigned to one of five habitat types.

We included 72 squirrel‐year pairs across 46 individual adult female GMGS in our habitat selection analyses (mean annual observations per squirrel = 88, range = 55–100, SD = 15); of these, we modeled density dependence for 64 squirrel‐year pairs (40 individuals), pre‐hibernation mass for 62 squirrel‐year pairs (40 individuals), annual survivorship for 61 squirrel‐year pairs (39 individuals), same‐year litter size for 58 squirrel‐year pairs (37 individuals), and next‐year litter size for 33 squirrel‐year pairs (23 individuals). Our habitat classifications across the study area revealed that available habitat reflected 52.0% dry meadow, 20.2% aspen, 17.3% willow, 6.8% spruce, and 3.7% wet meadow (Figure 1).

### 3.1 Habitat selection

**FIGURE 2:**
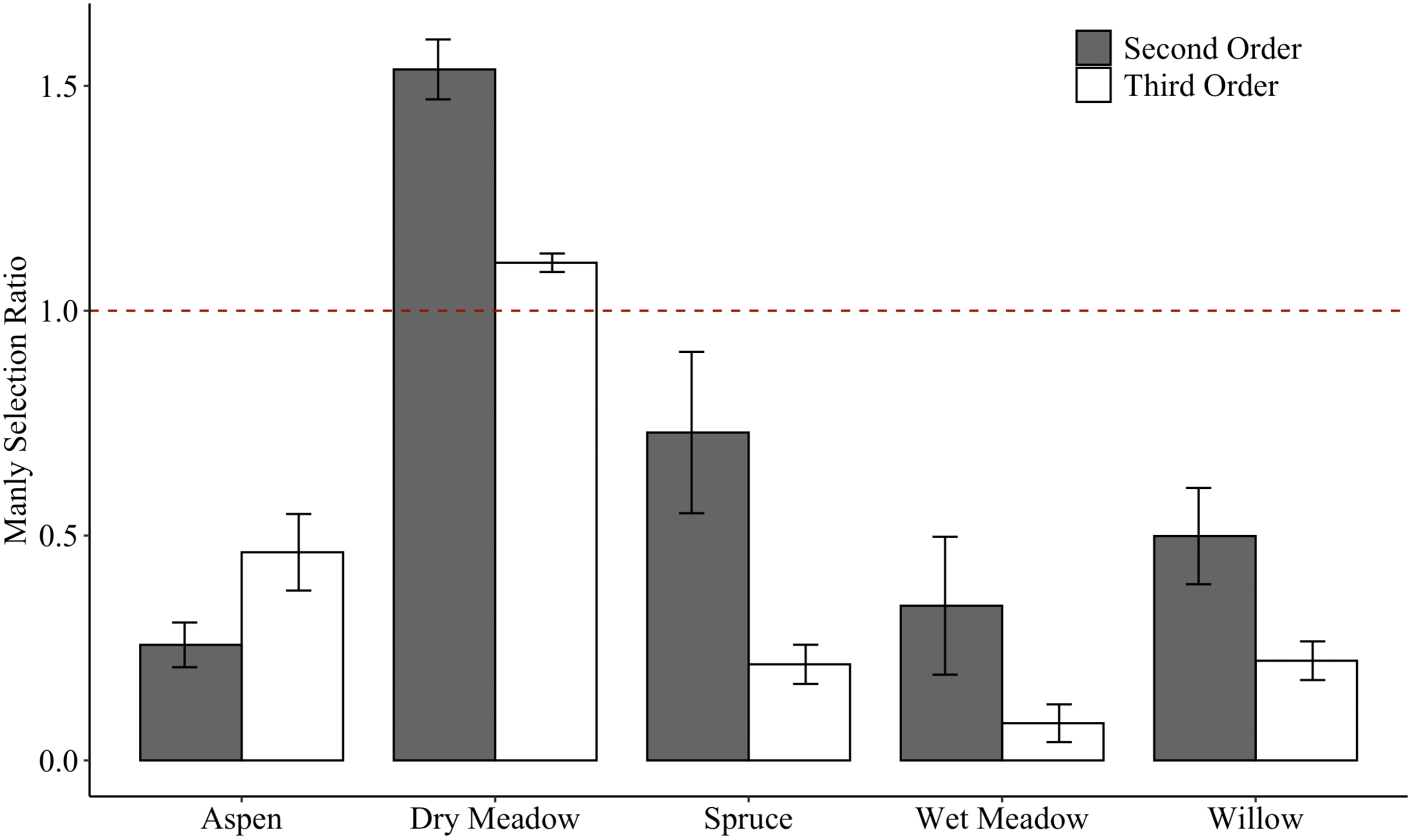
Mean selection ratios for second‐ and third‐order habitat selection by golden‐mantled ground squirrels across five macrohabitat types. Error bars represent standard errors. Values above the dashed line indicate positive selection and values below the dashed line indicate negative selection.

Macrohabitat use was non‐random at the spatial scale of both home‐range location (*p* < .0001, df = 288.0, χ^2^ = 7093.421) and use within the home range (*p* < .0001, df = 73.0, χ^2^ = 502.298). Manly selection ratios showed that GMGS demonstrated significant selection for dry meadow habitat, and selection against all other habitat types, at both spatial scales (Table S2, Figure 2).

**FIGURE 3:**
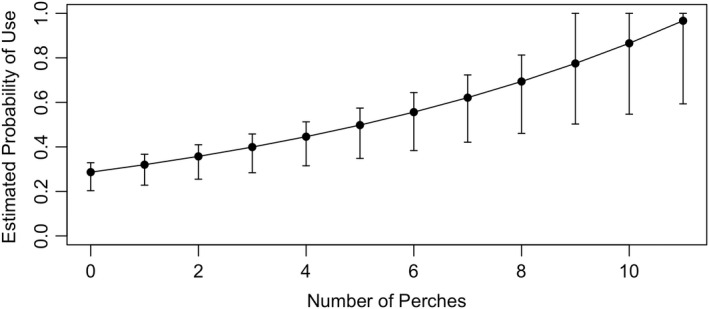
Prediction frame showing the estimated probability of use of a dry meadow grid square by golden‐mantled ground squirrels based on the number of perches (rocks or stumps) within that grid square. Error bars represent 95% confidence intervals from the probability distribution.

At the microhabitat level, the use of dry meadow was significantly related to the number of perches and to vegetation height (Table 1). As the number of perches within a dry meadow grid increased, squirrels were more likely to use that grid; a predictive plot based on our microhabitat model found that there was an 86.6% (±SE of 16.3%) probability of use of a grid containing 11 or more perches (Figure 3). Vegetation height was negatively related to the use of dry meadow once vegetation reached a height of 75 cm (category 4). Vegetation density was not included in our final model because it was positively associated with vegetation height (Pearson's Chi‐squared test, χ^2^ = 1693.70, df = 9, *p* < .0001).

**TABLE 1:** Model estimates from the analysis of number of observations in dry meadow habitat in relation to number of perches and vegetation height, after controlling for individual identity and year.

Model variables	Estimate	Standard error	*z* value	Pr (>|z|)
Intercept	−1.17	0.22	−5.38	<.0001
Number of perches	0.11	0.01	7.41	<.0001
Vegetation height (26–50 cm)	0.13	0.18	0.71	.475
Vegetation height (51–75 cm)	−0.13	0.18	−0.74	.459
Vegetation height (>75 cm)	−0.81	0.26	−3.07	.002

### 3.2 Density effects and fitness consequences

Population‐level density within our 16‐ha study area fluctuated more than fivefold during the study period (range = 5–29 adult females), and local density varied substantially as well (1–16 adult females). However, we found no relationship between population density and either percent of the home range comprised of dry meadow (GLMM β ± SE = 0.04 ± 0.03, *z* = 1.74, *p* = .081) or percent of observations within the home range that were in dry meadow habitat (GLMM β ± SE = −0.03 ± 0.02, *z* = −1.47, *p* = .141) (Table S3). Similarly, local density did not influence either the percent of the home range comprised of dry meadow (GLMM β ± SE = −0.01 ± 0.08, *z* = −0.15, *p* = .881) or the percent of observations within the home range that were in dry meadow (GLMM β ± SE = −0.07 ± 0.07, *z* = −1.00, *p* = .319) (Table S3). There was no relationship between age (yearling or older) and habitat use; percent home range comprised of dry meadow was not influenced by age when accounting for either population density (GLMM β ± SE = −0.09 ± 0.56, *z* = −0.16, *p* = .869) or local density (GLMM β ± SE = −0.13 ± 0.44, *z* = −0.28, *p* = .777), and habitat use was not affected by an interaction between age and either population density (GLMM β ± SE = 0.003 ± 0.03, *z* = 0.11, *p* = .912) or local density (GLMM β ± SE = 0.03 ± 0.10, *z* = 0.27, *p* = .789) (Table S3). Likewise, percent of observations within the home range that were in dry meadow habitat did not vary as a function of age when accounting for either population density (GLMM β ± SE = −0.99 ± 0.59, *z* = −1.67, *p* = .095) or local density (GLMM β ± SE = −0.22 ± 0.47, *z* = −0.47, *p* = .637), and there was no effect of an interaction between age and either population density (GLMM β ± SE = 0.05 ± 0.03, *z* = 1.68, *p* = .093) or local density (GLMM β ± SE = 0.05 ± 0.10, *z* = 0.48, *p* = .633) on within home‐range habitat use (Table S3).

Given that perches significantly increased the probability that GMGS will use dry meadow, we included total number of perches that each squirrel had in its home range as a third habitat predictor in our fitness models. We also included density as a covariate in all fitness models; because population density and local density were correlated (Pearson's correlation test, *r* = .67, df = 44, *p* < .0001), and because including correlated variables interferes with model performance, we used local density since it best reflects the density experienced by GMGS. Across years, mean percent survival (±SE) was 64.5 ± 7.7% (range 20–100). There was no relationship between annual survival and either percent home range comprised of dry meadow (GLMM β ± SE = −0.01 ± 0.37, *z* = −0.02, *p* = .982) or percent of observations within the home range that were in dry meadow habitat (GLMM β ± SE = −0.09 ± 0.36, *z* = −0.26, *p* = .794). However, we found that squirrels that selected for home ranges with a greater number of perches or that experienced lower local densities (fewer neighboring conspecifics within a given year) were significantly more likely to survive into the following year than squirrels that had access to fewer perches or experienced higher local densities (Table 2). Also, annual survival was significantly greater for yearlings than for older females (Table 2), an unexpected result that might reflect a survival cost of reproduction; yearlings are less likely to breed than older females (Kneip et al., 2011; Moore et al., 2016). Survival data for yearlings reflect those individuals that did not disperse; if a female decides to disperse it does so either during the year in which it is born (juveniles were not included in this study) or shortly after emerging from the first overwinter hibernation (these individuals did not meet sample size criteria and were excluded). Mean litter size (±SE) across years was 5.0 ± 0.2 pups (range 1–7). Litter size during the year that habitat use was recorded was not related to percent home range comprised of dry meadow (GLMM β ± SE = −0.10 ± 0.08, *z* = −1.39, *p* = .164), percent of observations within the home range that were in dry meadow (GLMM β ± SE = 0.04 ± 0.08, *z* = 0.58, *p* = .565), or the total number of perches within the home range (GLMM β ± SE = 0.00 ± 0.06, *z* = 0.01, *p* = .995). Similarly, litter size the next year was not related to percent home range comprised of dry meadow (GLMM β ± SE = 0.04 ± 0.13, *z* = 0.31, *p* = .756), percent of observations within the home range that were in dry meadow (GLMM β ± SE = 0.07 ± 0.11, *z* = 0.63, *p* = .531), or the number of perches within the home range (GLMM β ± SE = −0.07 ± 0.10, *z* = −0.75, *p* = .453). However, a bivariate analysis revealed a positive correlation between next‐year litter size and percent home range comprised of dry meadow that approached statistical significance (Pearson's correlation test, *r* = .32, df = 34, *p* = .055), although the relationship was not strong enough to appear in the final model. There was no relationship between pre‐hibernation mass and percent home range comprised of dry meadow (GLMM β ± SE = 0.03 ± 0.03, *z* = 1.28, *p* = .200), percent of observations within the home range that were in dry meadow (GLMM β ± SE = −0.02 ± 0.02, *z* = −1.10, *p* = .273), or number of perches in the home range (GLMM β ± SE = 0.00 ± 0.02, *z* = −0.17, *p* = .865).

**TABLE 2:** Model estimates from the analysis of annual survival in relation to number of perches within the home range, pre‐hibernation mass, local density, and age, after controlling for individual identity and year.

Model variables	Estimate	Standard error	*z* value	Pr (>|z|)
Intercept	−0.22	0.40	−0.54	.589
Number of perches	0.64	0.33	1.96	.050
Pre‐hibernation mass	0.60	0.32	1.85	.065
Local density	−0.63	0.30	−2.11	.035
Age (yearling)	1.41	0.65	2.18	.029

## 4 DISCUSSION

Habitat associations of golden‐mantled ground squirrels are not well known, but available information suggests the species is a generalist, occurring in a variety of forested or sparsely brushy habitats, and also on rocky slopes adjoining grasslands and at the margins of mountain meadows (Bartels & Thompson, 1993). A common feature of habitats used by GMGS appears to be the presence of some degree of openness. We found that GMGS exhibited strong selection for dry meadow habitat, and that pattern was consistent for both home‐range placement (second‐order selection) and use within the home range (third‐order selection). Dry meadow offers an open landscape and also contains an abundance of dandelions, a preferred food resource for GMGS in our study area (Carleton, 1966). Other species of ground‐dwelling squirrels also prefer open areas that provide good visibility, likely to facilitate early detection of predators (Blumstein et al., 2006; Ordeñana et al., 2012; Zaharia et al., 2016); the juxtaposition of these two conditions—food availability and predator detectability—seems to influence habitat selection decisions (Armitage, 2014; Hannon et al., 2006; McGrann et al., 2014). At our study site, GMGS coexist with chipmunks (*Tamias minimus*) and yellow‐bellied marmots (*Marmota flaviventer*); all three species are readily visible, and we have detected no competition for space.

Use of dry meadow by GMGS was promoted by the presence of perches and reduced vegetation height, highlighting the importance of a lack of visual obstruction in habitat selection decisions. Perches may abate predation risk by elevating a squirrel's point of view, enabling visual monitoring of the surroundings (Armitage, 1982; Beauchamp, 2015). Most of the perches in our study were rocks; GMGS were found associated with rocks in other studies (Bartels & Thompson, 1993; Bihr & Smith, 1998). Prominences such as rocks have been associated with vigilance behavior in several other species of ground‐dwelling squirrels (Leger et al., 1983; Machutchon & Harestad, 1990; Tyser, 1980). Similar to presence of perches, reduced vegetation height likely reduces predation risk by enhancing detection of predators. Our results are consistent with those of Rowe (2007), who found that GMGS in Utah preferred grazed areas to ungrazed areas. Studies on other species of ground‐dwelling squirrels, such as yellow‐bellied marmots (Bednekoff & Blumstein, 2009; Van Vuren, 2001), thirteen‐lined ground squirrels (*Ictidomys tridecemlineatus*; Arenz & Leger, 1997), Great Basin ground squirrels (*Urocitellus mollis*; Sharpe & Van Horne, 1998), Arctic ground squirrels (*Urocitellus parryii*; Wheeler & Hik, 2014), and alpine marmots (*Marmota marmota*; Ferrari et al., 2009), also suggest that visual obstruction can increase predation risk. In addition to aiding in predator detection directly, perches may indirectly promote predator detection through the monitoring of conspecifics; even in asocial species, vigilance of neighbors may be interpreted as public information about present risk (Beauchamp, 2017; Carrasco & Blumstein, 2011; Sirot, 2006).

GMGS often occur in open coniferous forests (McKeever, 1964), but individuals avoided spruce forest in our study, perhaps because spruce forest had a closed canopy that was perceived as being too dense; GMGS in a mature conifer forest in Montana preferred locations with a reduced canopy tree density (Shick et al., 2006). Wet meadow and willow habitats also were avoided by GMGS; in both habitats vegetation was exceptionally dense, creating visual obstruction, and damp soils in these habitats might have been unsuitable for excavating burrows. GMGS in Utah preferred xeric habitats to mesic habitats (Rowe, 2007), perhaps because xeric habitats support sparser vegetation. Avoidance of wet meadow and willow habitats also might result from an observation bias in data collection; squirrels were readily observed in dry meadow, spruce, and aspen habitats, but not in wet meadow or willow because of visual obstruction for the observer. However, this observation bias likely is minor; we seldom observed squirrels entering wet meadow or willow habitats, and when they did, they usually traversed the habitat patch and exited on the other side.

We found no effect of density on use of dry meadow, the preferred habitat, either for home‐range location (second‐order selection) or for occurrence within the home range (third‐order), despite substantial variation in both population‐level and local‐level density. As population density or local density increased, squirrels stayed in dry meadow habitat rather than moving to less crowded, presumably marginal habitat, contrary to the expectations of IFD. For resident adult females with established home ranges, relocation in response to high density may have been too costly. However, the cost should be lower for yearlings, who are making the decision of where to settle; hence, we were surprised to find no density effect for yearlings. It is possible that the lack of spillover into marginal habitats as density increased resulted because available marginal habitats were not of sufficient quality, or there was enough dry meadow to accommodate squirrels at high density; all habitats besides dry meadow were avoided. Further, home ranges did not decrease in size during high‐density years (Aliperti, 2020). Regardless of the cause for a lack of spillover, with increasing density female GMGS continued to preferentially select for dry meadow habitat, suggesting the potential for increased competition. Accordingly, we found that local density had a significant, negative effect on survival, upholding the IFD prediction that increased crowding into patches of preferred habitat should decrease fitness (Fretwell & Lucas, 1969). We did not find a similar effect at the population level. Individual squirrels tended to restrict their activities to one of several clusters within the study area (Wells & Van Vuren, 2017); for organisms with relatively limited mobility, density effects may be exhibited at scales less than that of the population (Einum & Nislow, 2005).

High‐quality habitat might confer increased fitness through resources related to either food availability or predation risk. Reproduction might be enhanced due to greater availability or quality of food resources (Kenagy et al., 1990; Wells & Van Vuren, 2018), and survival might be improved due to increased fat stores before hibernation or reduced predation risk (Dark, 2005; Kneip et al., 2011). However, we did not find an effect of use of dry meadow on reproduction, although a marginally significant correlation between dry meadow use and next‐year litter size suggests that such an effect was a possibility, which would support the notion that GMGS are capital breeders (Stearns, 1992). Similarly, we did not find a relationship between dry meadow use and pre‐hibernation mass or overwinter survival. However, we found that GMGS with more perches in their home range experienced higher survival, substantiating a link between enhanced visibility and decreased predation risk. Hence, the value of dry meadow habitat to GMGS may lie more in reduced predation risk than in enhanced food availability.

Our results show that female golden‐mantled ground squirrels strongly preferred dry meadow habitat and, contrary to the expectations of IFD, they maintained that affinity as density increased, perhaps because marginal habitats of sufficient quality were not available. The main fitness benefit of dry meadow appeared to be reduced predation risk, and evidently the benefit of reducing predation risk outweighed the survival cost of competing for resources in a crowded environment. Given that alterations to habitat may influence the spatial distribution of both individuals and preferred resources, understanding the causes and consequences of multi‐scale habitat use provides insight to the fields of wildlife management and conservation.

## AUTHOR CONTRIBUTIONS

**Jaclyn Aliperti:** Conceptualization (supporting); data curation (equal); formal analysis (lead); funding acquisition (supporting); investigation (equal); methodology (equal); writing – original draft (lead). **Kimberly Jenderseck:** Conceptualization (supporting); investigation (supporting); methodology (supporting). **Dirk Van Vuren:** Conceptualization (lead); data curation (equal); formal analysis (supporting); funding acquisition (lead); investigation (equal); methodology (equal); project administration (lead); resources (lead); supervision (lead); writing – review and editing (lead).

## Supporting information


Table S1‐S3

## Data Availability

The data that support the findings of this study have been archived in a publicly accessible repository on Dryad, available at: https://doi.org/10.25338/B8T34G.
