# Supplementary material for: Habitat selection in a fluctuating ground squirrel population: Density‐dependence and fitness consequences
Source: Ecol Evol. 2022 Aug 29;12(8):e9241. doi: 10.1002/ece3.9241 (PMC9424181; doi:10.1002/ece3.9241)
Supplement: Supplementary file 1 — Table S1‐S3 [file ECE3-12-e9241-s001.docx]

**Table S1.** Model selection table showing fixed candidate variables and AICc values for all fitness models. All models accounted for individuals and years of study as random effects. PHM = pre-hibernation mass; EM = emergence mass; %HR = percent home range comprised of dry meadow; %OBS = percent of observations within HR in dry meadow; perches = total number of perches in the home range.

| **Outcome** | **Fixed Candidate Variables** | **AICc** | **Δ AICc** | **logLik** | **Residual df** |
| --- | --- | --- | --- | --- | --- |
| Annual Survival | PHM + perches + local density + age | 84.8 | 0.0 | -34.4 | 54 |
|  | PHM + %OBS + perches + local density + age | 87.4 | 2.6 | -34.3 | 53 |
|  | PHM + %HR + perches + local density + age | 87.4 | 2.6 | -34.3 | 53 |
|  | PHM + %HR + %OBS + perches + local density + age | 90.1 | 5.3 | -34.3 | 52 |
| Same-year Litter Size | EM | 229.7 | 0.0 | -110.5 | 54 |
|  | EM + %HR | 230.5 | 0.8 | -109.7 | 53 |
|  | EM + %HR + age | 232.3 | 2.6 | -109.3 | 52 |
|  | EM + %HR + %OBS + age | 234.4 | 4.7 | -109.1 | 51 |
|  | EM + %HR + %OBS + local density + age | 236.8 | 7.1 | -108.9 | 50 |
|  | EM + %HR + %OBS + perches + local density + age | 239.6 | 9.9 | -108.9 | 49 |
| Next-year Litter Size | %OBS | 135.0 | 0.0 | -62.8 | 29 |
|  | %OBS + perches | 137.2 | 2.2 | -62.5 | 28 |
|  | %HR + %OBS + perches | 140.2 | 5.2 | -62.5 | 27 |
|  | %HR + %OBS + perches + local density | 143.3 | 8.3 | -62.4 | 26 |
|  | %HR + %OBS + perches + local density + age | 146.8 | 11.8 | -62.4 | 25 |
|  | PHM + %HR + %OBS + perches + local density + age | 150.7 | 15.7 | -62.4 | 24 |
| Pre-hibernation Body Mass | %HR | -60.6 | 0.0 | 35.8 | 57 |
|  | %HR + %OBS | -59.7 | 0.9 | 36.6 | 56 |
|  | %HR + %OBS + local density | -57.3 | 3.3 | 36.7 | 55 |
|  | %HR + %OBS + local density + age | -54.7 | 5.9 | 36.7 | 54 |
|  | %HR + %OBS + perches + local density + age | -52.0 | 8.6 | 36.7 | 53 |

**Table S2.** Output table of Manly selection ratios for second-order habitat selection (home range placement) and third-order habitat selection (use within the home range). Proportion of habitat available and used is shown as a population average for second-order selection. Habitat availability was unique to each individual for third-order selection. Wi = selection ratio; SE = standard error; IC_lower_ = 5% confidence interval limit; IC_upper_ = 95% confidence interval limit.

|  | **Habitat Type** | **Available** | **Used** | **Wi** | **SE** | **IC_lower_** | **IC_upper_** |
| --- | --- | --- | --- | --- | --- | --- | --- |
| Second-order | Aspen | 0.2019 | 0.0519 | 0.2570 | 0.0497 | 0.1289 | 0.3850 |
|  | Dry Meadow | 0.5204 | 0.7996 | 1.5366 | 0.0666 | 1.3651 | 1.7082 |
|  | Spruce | 0.0679 | 0.0495 | 0.7291 | 0.1794 | 0.2669 | 1.1913 |
|  | Wet Meadow | 0.0367 | 0.0126 | 0.3440 | 0.1533 | -0.0510 | 0.7390 |
|  | Willow | 0.1731 | 0.0864 | 0.4989 | 0.1071 | 0.2231 | 0.7746 |
| Third-order | Aspen  Dry Meadow  Spruce  Wet Meadow  Willow | -  -  -  -  - | -  -  -  -  - | 0.4628  1.1067  0.2136  0.0827  0.2217 | 0.0851  0.0205  0.0436  0.0420  0.0430 | 0.2436  1.0538  0.1014  -0.0255  0.1110 | 0.6821  1.1596  0.3259  0.1909  0.3324 |

**Table S2.** Model estimates showing effects of density on the proportion of dry meadow habitat in the home range (second-order selection) and on the proportion of observations within the home range within dry meadow habitat (third-order selection). Presented are outputs from separate models for each level of density (population and local) at each spatial scale (second-order and third-order). Age was a binary variable that categorized each squirrel as being either a yearling or older. All models were logistic GLMMs with a binomial error distribution and logit link that weighted the outcome by the total number of grid squares in a squirrel’s home range (second-order model) or the total number of observations recorded for each squirrel (third-order model) and that included individuals and years as random effects. An asterisk (*) denotes an interaction between variables. See text for details.

| **Outcome** | **Fixed Model Variables** | **Estimate** | **Standard Error** | **z value** | **Pr(>\|z\|)** |
| --- | --- | --- | --- | --- | --- |
| Percent home range comprised of dry meadow | Intercept | 1.57 | 0.51 | 3.10 | 0.002 |
|  | Age (yearling) | -0.09 | 0.56 | -0.16 | 0.869 |
|  | Population density | 0.04 | 0.03 | 1.74 | 0.081 |
|  | Age(Yearling)*Population density | 0.003 | 0.03 | 0.11 | 0.912 |
| Percent home range comprised of dry meadow | Intercept | 2.28 | 0.43 | 5.35 | <0.0001 |
|  | Age (yearling) | -0.13 | 0.44 | -0.28 | 0.777 |
|  | Local density | -0.01 | 0.08 | -0.15 | 0.881 |
|  | Age(Yearling)*Local density |  |  |  |  |
| Percent observations in home range within dry meadow | Intercept | 4.23 | 0.45 | 9.50 | <0.0001 |
|  | Age (yearling) | -0.99 | 0.59 | -1.67 | 0.095 |
|  | Population density | -0.03 | 0.02 | -1.47 | 0.141 |
|  | Age(Yearling)*Population density | 0.05 | 0.03 | 1.68 | 0.093 |
| Percent observations in home range within dry meadow | Intercept | 4.04 | 0.38 | 10.72 | <0.0001 |
|  | Age (yearling) | -0.22 | 0.47 | -0.47 | 0.637 |
|  | Local density | -0.07 | 0.07 | -1.00 | 0.319 |
|  | Age (yearling)*Local density | 0.05 | 0.10 | 0.48 | 0.633 |
